# Developing a Silk Fibroin Composite Film to Scavenge and Probe H_2_O_2_ Associated with UV-Excitable Blue Fluorescence

**DOI:** 10.3390/s20020366

**Published:** 2020-01-08

**Authors:** Tze-Wen Chung, Chun-Yi Chang, Chun-Ning Chang, Chiu-Hsun Liao, Yun-Jen Jan, Li-Ting Chen, Weng-Pin Chen

**Affiliations:** 1 Department of Biomedical Engineering, National Yang-Ming University, No.155, Sec.2, Linong Street, Taipei 11221, Taiwan; 30304016@gm.ym.edu.tw (C.-Y.C.); ase830217@gmail.com (C.-N.C.); liting0515@gmail.com (L.-T.C.); 2 The Center for Advanced Pharmaceutics and Drug Delivery Research, National Yang-Ming University, Taipei 11221, Taiwan; 3 Miaoli District Agricultural Research and Extension Station, Council of Agriculture, Executive Yuan, Miaoli County 36346, Taiwan; jsliaw@mdais.gov.tw (C.-H.L.); or; 4 Department of Mechanical Engineering, National Taipei University of Technology, Taipei 10608, Taiwan; 5 Additive Manufacturing Center for Mass Customization Production, National Taipei University of Technology, Taipei 10608, Taiwan

**Keywords:** silk fibroin, graphene oxide, H_2_O_2_ probe, H_2_O_2_ scavenge, blue fluorescence

## Abstract

A silk fibroin composite film that can simultaneously scavenge and probe H_2_O_2_ in situ was developed for possibly examining local concentrations of H_2_O_2_ for biomedical applications. A multi-functional composite film (GDES) that consists of graphene oxide (G), a photothermally responsive element that was blended with polydopamine (PDA, D)/horseradish peroxidase (HRP, E) (or DE complex), and then GDE microaggregates were coated with silk fibroin (SF, S), a tyrosine-containing protein. At 37 °C, the H_2_O_2_-scavenging ability of a GDES film in solution at approximately 7.5 × 10^−3^ μmol H_2_O_2_/mg film was the highest compared with those of S and GS films. The intensities of UV-excitable blue fluorescence of a GDES film linearly increased with increasing H_2_O_2_ concentrations from 4.0 μM to 80 μM at 37 °C. Interestingly, after a GDES film scavenged H_2_O_2_, the UV-excitable blue fluorescent film could be qualitatively monitored by eye, making the film an eye-probe H_2_O_2_ sensor. A GDES film enabled to heat H_2_O_2_-containing samples to 37 °C or higher by the absorption of near-IR irradiation at 808 nm. The good biocompatibility of a GDES film was examined according to the requirements of ISO-10993-5. Accordingly, a GDES film was developed herein to scavenge and eye-probe H_2_O_2_ in situ and so it has potential for biomedical applications.

## 1. Introduction

H_2_O_2_, a reactive oxygen species (ROS), participates in numerous physiological and pathological conditions, including stem cell proliferation and differentiation, anti-bacterial defense, wounds, cancer, and ageing [1,2]. The concentration of H_2_O_2_ in vivo affects cellular responses. For example, at a low concentration (10^−2^~10^−1^ μM), H_2_O_2_ promotes the proliferation of stem cells while at a medium to high concentration (10^0^~10^2^ μM), it arrests cell growth and consequently causes the apoptosis of cells, as in the ageing process [1,2]. Moreover, the production of H_2_O_2_ is one of three important early signals that indicate damage in response to tissue injury or inflammation [2,3,4]. However, current research focuses on antioxidant therapy using antioxidants such as Medihoney^®^ [5] or the probing of H_2_O_2_ using cytotoxic organic dyes, such as penta-fluoro-benzenesulfonyl-fluorescein [6]. Hence, developing a medical sensing device that can both scavenge and probe the concentration of local H_2_O_2_ is important for the clinical management of varying pathological conditions, including decubital ulcers or non-healing diabetic foot [2]. This work develops an SF composite film with both H_2_O_2_-scavenging and H_2_O_2_-probing properties for possible biomedical use.

Graphene oxide (GO or G) is a two-dimensional carbon-containing material that is produced by the exfoliation of graphite. GO has a high specific surface area and contains abundant hydrophilic moieties including carboxyl groups, hydroxyl groups, and epoxy groups on its surface [7]. Owing to its photothermal conversion and many functional groups, GO is extensively used in the photothermal treatment of cancers [8,9], and in gene/drug carriers for DNA or proteins as it forms hydrogen bonds and/or undergoes π-π stacking interactions with its cargo [9,10].

Horseradish peroxidase (HRP or E) is a plant-derived enzyme that is widely used in the oxidative polymerization of phenolic compounds in the presence of H_2_O_2_ owing to its catalysis of the reduction of H_2_O_2_. HRP was frequently employed in the detection of H_2_O_2_ and in biosensors such as glucose biosensors in which it is combined with glucosidase [11,12,13], Amplex^®^ Red [14] and 3,5,3′,5′-tetramethylbenzidine (TMB) [15] are HRP-based H_2_O_2_ detection agents for biochemical analysis in the laboratory. To fabricate HRP-based H_2_O_2_ biosensors, HRP can be immobilized on various substrates, including SF, L-dopamine (DA) matrix, and silica macropores [12,16,17].

Polydopamine (PDA or D) is a polymer of DA which contains catechol and primary amine moieties [18] and has a variety of applications. For instance, PDA nanoparticles that were produced by Ju et al. could scavenge free radicals, including H_2_O_2,_ perhaps because of its catechol/quinone structure [19]. Melanin-like PDA nanoparticles that were fabricated by Liu et al. exhibited both H_2_O_2_ -scavenging ability and a photothermal conversion efficiency that exceeds that of gold nanorods [20]. Moreover, since PDA is a highly adhesive polymer, it can be easily immobilized or blended with various enzymes to form PDA/enzyme complex [18]. The adhesive property of PDA was exploited herein by blending HRP with PDA to produce PDA-HRP (DE) complexes in solutions.

Silk fibroin (SF or S) was extracted from *Bombyx mori* cocoons after a degumming process. It was extensively examined for use in tissue engineering [21] and drug delivery [22]. Approximately 5% SF in molar amount is tyrosine moiety, which is one of phenolic compounds that can be oxidized by HRP/H_2_O_2_ to produce dityrosine bonds, resulting in the crosslinking of SF to form an SF network or hydrogel [23]. Interestingly, UV irradiation of a large amount of dityrosine bonds that form in SF networks or hydrogels causes the emission of blue fluorescence [23]. The intensity of blue fluorescence from SF hydrogels that are excited by UV irradiation may be monitored using a fluorimeter to quantify H_2_O_2_ in solution, and it can be observed by the naked eye, enabling such hydrogels to be used eye-probes of H_2_O_2_.

**Figure 1:**
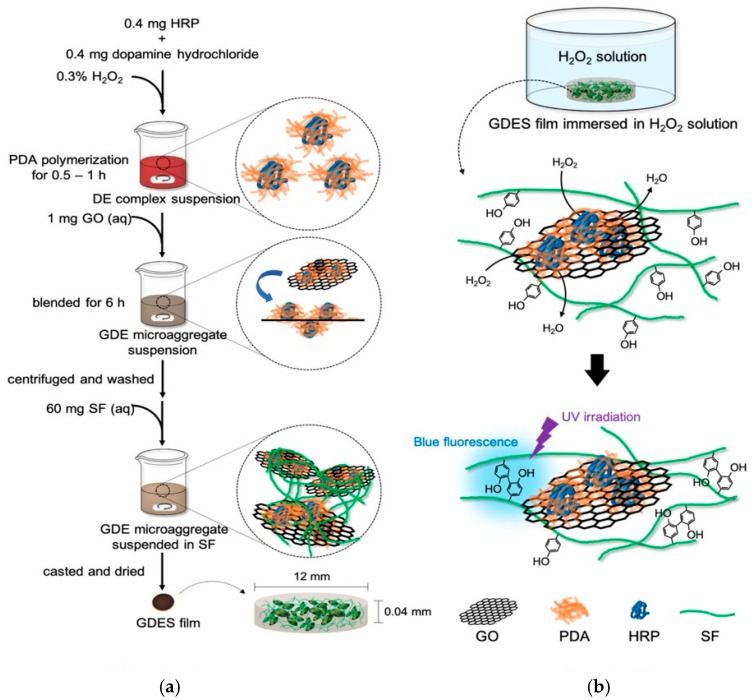

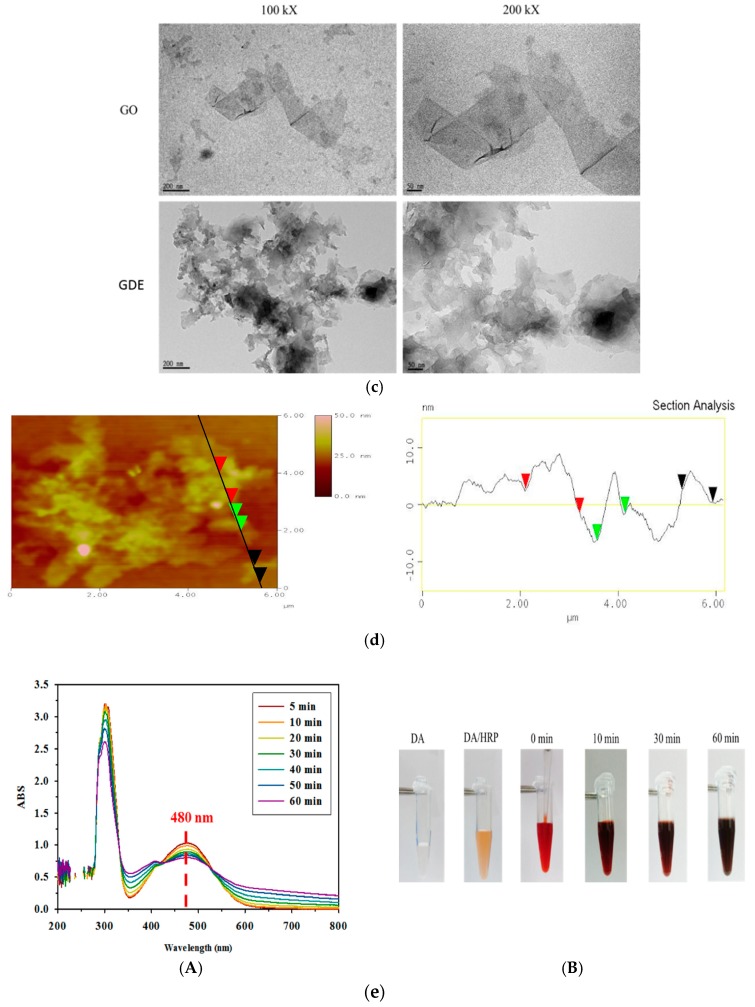
(**a**) Schematic figure for fabricating GDES films, (**b**) Schematic figure for the principle of scavenging/probing H_2_O_2_ by GDES films. (**c**) TEM micrographs for GO and GDE microaggregates were shown. (**d**) The AFM image for GDE microaggregates was shown. (**e**) polymerization of dopamine to PDA catalyzed by HRP. (**A**) UV-Vis absorbance kinetics; (**B**) color changes during PDA formation. Absorbance at 480 nm, which correlates to the red intermediate during polymerization decrease over time.

A multi-functional composite GDES film was developed to scavenge and probe H_2_O_2_ with UV-excitable blue fluorescence in aqueous solution. The film consists of GO, HRP, PDA and SF, and its fabrication is schematically depicted in Figure 1a.

## 2. Materials and Methods

Graphite was purchased from Alfa Aesar (325 mesh, Thermo Fisher Scientific, Waltham, MA, USA). *Bombyx mori* cocoons were provided by the Miaoli District Agricultural Research and Extension Station, Council of Agriculture, Executive Yuan of the ROC. H_2_O_2_ (30%) was purchased from Merck Millipore (Billerica, MA, USA). HRP (Type VI, ≥250 units/mg solid, MW ~ 44.1 kDa), dopamine hydrochloride (MW = 153.18 Da), 1,10-phenanthroline (MW = 180.2 Da), potassium permanganate (MW = 158.03 Da), sodium nitrate (MW = 84.99 Da), sodium nitrite (MW = 68.99 Da), and sodium molybdate (MW = 205.92 Da) were bought from Sigma Aldrich (St. Louis, MO, USA). An MTS assay kit for in the biocompatibility test was purchased from Promega (Madison, WI, USA). The Bradford reagent for the protein assay was purchased from Bio-RAD Corp (Hercules, CA, USA).

### 2.1. Fabrication of GDES Films

Graphene oxide (GO) was synthesized using a modified Hummer’s method [24]. Briefly, 1 g graphite flakes and 0.5 g NaNO_3_ were mixed with concentrated (98%) sulfuric acid, and 3 g KMnO_4_ was slowly added to the mixture at 0 °C. The mixture was vigorously stirred for 24 h at 35 °C. The reaction was quenched by adding DI water and H_2_O_2,_ and also to remove those oxidation agents and the GO containing suspension was combined with HCl. Finally, GO/solvent suspensions were washed, centrifuged several times to remove solvents, and GO in the bottom containing residual solvents was evaporated off at 50 °C to produce dry GO.

To synthesize PDA-HRP (or DE) complexes, a modified method of Dai et al. [16] was adopted to synthesize PDA-HRP (or DE) complexes. Briefly, 0.4 mg HRP and 0.4 mg DA (1:1 in wt.) were dissolved in PBS (Phosphate Buffered Saline), and 0.3% H_2_O_2_ was added to the DA/HRP mixture to promote the polymerization of DA. The polymerization of PDA in the mixture rapidly changed the color of the mixture from none (or transparent) to dark red. The reaction was continued for 0.5–1 h to prevent over-polymerization of PDA. Since HRP is an enzyme with a large molecular weight (~44 KDa), the PDA was most likely located on the surface of HRP (Figure 1a).

To prepare GDE microaggregates, 1 mg GO that was dispersed in 1.5 mL DI water was added to dark red DE suspensions and vigorously stirred, causing DE complexes to adhere to the GO surface, forming GDE microaggregates. The GDE microaggregates, containing suspensions, were centrifuged and washed several times to remove non-adhering DE complexes. The particle sizes of the GDE aggregates were measured and checked using a laser particle size analyzer (Particulate Systems, Nano-Plus, Norcross, GA, USA).

SF (MW~127 kDa) solution was prepared as described in early reports that were published by the authors’ laboratory [25]. Briefly, *B. mori* cocoons were boiled with 0.02% Na_2_CO_3_ to degum to obtain SF. SF was dissolved in 9.3 M LiBr solution, and then dialyzed in DI water to remove Li^+^ to produce 5~10 wt% of SF solution for further applications. To prepare GDES films, 60 mg of the aforementioned SF solution was added to GDE micro-aggregate suspension and stirred to cover GDE microaggregates completely with SF, producing GDES microaggregates in suspension. Aliquots of the suspensions were extracted and evaporated at 45 °C to produce a dry GDES film with size of 12 mm (diameter) × 0.04 mm (thickness) and about 12.32 mg (Figure 1a) with the ratios of compositions of G:D:E:S of 1.62%:0.62%:0.39%:97.4%, respectively. The films were further treated by varying percentages of ethanol (e.g., 75%) for about 15 min to induce β-sheets of SF (e.g., ~45%) to stabilize the SF polymers in solutions [26].

### 2.2. Characterizations of Components and Films Using ATR-FTIR, SEM and TEM

Attenuated total reflectance Fourier transform infrared spectra (ATR-FTIR) of various samples (i.e., GO, PDA, HRP, SF, DE, GDE, and GDES) were examined using an ATR-FTIR spectrometer (IRAffinity-1, Shimadzu, Japan). The samples were scanned from 4000 cm^−1^ to 600 cm^−1^ at a resolution of 4 cm^−1^ and the spectra that were obtained from the IRsolution software were baseline-corrected and smoothed.

Squares of the GDES films with an area of 1.5 cm^2^ were cut and their surface morphologies were examined via scanning electron microscopy (SEM) (JSM-7600F, JEOL, Tokyo, Japan) following procedures that was previously employed by the authors’ group [26]. Briefly, the films were immersed in 80 μM H_2_O_2_, so-called H_2_O_2_-treated GDES, and in PBS, GDES film, respectively, for 30 min at 37 °C, dried and coated with platinum for SEM. The details of the procedure can be found elsewhere [25]. The morphology of GO was observed by transmission electron microscopy (TEM, JEM-2000EXII, JEOL, Tokyo, Japan) according to a procedure that was previously used by the authors’ group [26,27]. The particle sizes of the GO and GDE microaggregates were determined using a zeta potential/nanoparticle analyzer (Nano-Plus Particulate System, Norcross, GA, USA). The images of the surfaces of GDE microaggregates were taken by a tapping mode with a Si cantilever (App Nano, ACSTA-50, Mount View, CA, USA) by an atomic force microscope (AFM) (Bruker, Dimension Icon, Billerica, MA, USA) to examine the roughness of the surfaces equipped with built-in software (Nanoscope IIIa, Digital Instrument, Santa Barbara, CA, USA) [26].

To determine the E and D contents of the GDE microaggregates, the Bradford protein assay [28] for HRP quantification and the Arnow assay [29] for determining the catechol contents of PDA were carried out on the supernatant after the GDE suspension had been centrifuged. For the Bradford protein assay, 1.6 mL of the Bradford reagent was added to 0.4 mL of supernatant and allowed to react for 10 min; then absorbance at 595 nm was measured using a UV/VIS spectrophotometer (Multiskan, Thermo Scientific, Waltham, MA USA). To perform the Arnow assay, 1 mL of supernatant was mixed with 1 mL of 0.5 M HCl, 1 mL of a mixed sodium nitrite/sodium molybdate solution, 1 mL of 1 M NaOH and 1 mL of DI water, and the absorbance at 510 nm was measured.

### 2.3. H_2_O_2_ Scavenging Assay

An H_2_O_2_ solution with a concentration of 64 μM was applied to the as-prepared GDES and GS films to measure the H_2_O_2_-scavenging abilities of the films at various temperatures of 15 °C, 25 °C, or 37 °C. The residual H_2_O_2_ in the solution after reaction with the films was pipetted out for analysis by the 1,10-phenanthroline/FeCl_2_ method [30,31]. In the absence of H_2_O_2_, 1,10-phenanthroline molecules form chelates with ferrous ions (Fe^2+^), and these chelates exhibit a significant absorption peak at 510 nm. In contrast, in the presence of H_2_O_2_, the Fenton reaction takes place, transforming the ferrous ions (Fe^2+^) of FeCl_2_ to ferric ions (Fe^3+^). Accordingly, the chelate of 1,10-phenanthroline molecules/Fe^2+^ does not form and no absorption peak at 510 nm is detected. The degrees of absorption at 510 nm by the pipetted supernatant solutions on the SF, EDG and EDGS films were determined using a UV/VIS spectrophotometer. The H_2_O_2_-scavenging abilities of the films were determined by subtracting the absorption value at 510 nm by the highest concentration of H_2_O_2_ on the calibration line from the absorption value at 510 nm by those pipetted supernatant solutions.

To determine the H_2_O_2_-scavenging ability of the formed dityrosine and the auxiliary photothermal conversion ability of PDA, the graphene oxide/silk fibroin (GS) film was made in a manner similar to the control film, which was fabricated from GO and SF.

### 2.4. Probing H_2_O_2_ by Measuring UV-Excitable Blue Fluorescence of GDES Film

To probe H_2_O_2_ by examining the emission of the UV-excitable blue fluorescence of GDES films, such films were immersed in varying concentrations of H_2_O_2_ solutions to allow the immobilized HRP to crosslink tyrosine to dityrosine bonds in SF of the films. After 30 min of reaction, the intensity of blue fluorescence in the solutions in test wells was relatively stable and could be observed by the naked eye under UV irradiation. The intensity of the fluorescence of the suspensions was determined by measuring the intensity of emission wavelength at 425 nm with bandwidth 20 nm following irradiation by an excitation wavelength of 325 nm with bandwidth of 9 nm using a fluorescent spectrophotometer equipped with Xenon flash lamp (Infinite 200, Tecan, Männedorf, Switzerland) at 37 °C (shown in supplement (Figure S1)). The data were further analyzed using Origin 8 (Origin Lab, Northampton, MA, USA).

### 2.5. Photothermal Conversion of GDES Films

Carbonaceous materials such as graphene oxide and carbon nanotubes are effective agents of photothermal conversion [8]. They convert energy that is absorbed in the form of infrared light into heat; PDA materials reportedly have the same property [20]. The photothermal conversion effects of GDES and GS films in 2 mL H_2_O were assessed by irradiating them using a 2W 808 nm NIR [20], and continuously measuring the temperatures of the solutions using a K-type thermocouple with resolution of 0.1 °C at temperature range of this study for 10 min.

### 2.6. Assessment of In Vitro Biocompatibility

The in vitro biocompatibility of GDES film was determined according to standards of ISO 10993-5 and 10993-12 [32]. L929 fibroblasts were purchased from the Bio-resource Collection and Research Center (BCRC, Hsin-Chu, Taiwan) and cultured in Dulbecco’s modified Eagle’s medium that contained 10% horse serum at 37 °C in a 5% CO_2_ incubator. To obtain various dilutions of extraction supernatant solutions on the GDES films, the films were immersed into a culture medium, for 24 h, which was then diluted with a fresh culture medium to make different diluted ratios of mediums. According to requirements of ISO-10993-5, the L929 fibroblasts were then incubated with the various extraction of the various diluted-ratio mediums. for another 24 h, and an MTS assay ((3-(4,5-Dimethylthiazol-2-yl)-5-(3-carboxymethoxyphenyl)-2-(4-sulfophenyl)-2-tetrazolium) and MW. of 487.5 Da, abcam plc, Cambridge, UK), a colorimetric method, was performed to quantify cell proliferation and viability, according to the manufacturer’s instructions. The cell viability of each group was compared with that of the control group in which no extraction medium was used.

### 2.7. Statistical Analysis

A Student’s *t* test was conducted to analyze the statistical significance of the variations in the H_2_O_2_-scavenging ability among SF, GS, and GDES films at each working temperature, and among GDES films at various working temperatures. A confidence level of 95% was used to determine statistical significance. Data are presented as mean ± SD from triplicate measurements.

## 3. Results and Discussion

### 3.1. Fabricating GDES Films and Analysis of Their Compositions

The lateral size of GO that was prepared using the modified Hummer’s method was in the range of 200~500 nm, as determined from TEM micrographs (Figure 1c). Also, the lateral size of GDE (Table 1) aggregates around several μm (e.g., 3–5 μm) were shown (Figure 1c). In addition, the sizes of GO and GDE aggregates were 279 ± 8 nm and 1692 ± 13 nm (n = 3), respectively, as determined using a zeta/nanoparticle analyzer. The size of GDE microaggregates was around 1.7 μM, possibly because DE adhered to the GO surfaces, forming aggregates, perhaps because of extensive hydrogen bonding and π-π interactions between PDA and GO [7,33,34]. However, the possibly forming aggregates by GO during adhesion of DE to GO surfaces could not be ruled out although the suspensions were prepared in highly stirring conditions. To determine the E and D contents of GDE microaggregates, the Bradford protein assay for HRP and the Arnow assay for determining the catechol content of PDA were conducted [28,29]. The results revealed that the amounts of E and D in GDE microaggregates were 240 μg and 380 μg per mg GO, respectively. Approximately 60% of the initial amount of E and 95% of that of D that was used in preparing the DE complexes adhered to the GO surfaces. Interestingly, the mass of DE in 1 mg GDE microaggregates (with a size of 1.7 μm) herein was around 620 μg, which was similar to those obtained elsewhere [35,36]. For instance, Xu and Lai obtained 600 μg and 300 μg of enzyme per mg GO, respectively [35,36]. Since the chemical structures of PDA and GO contains many aromatic rings and abundant hydrophilic moieties, including carboxyl groups and hydroxyl groups [7,33,34], 620 μg of DE complexes might have bonded to GO per gm, producing GDE microaggregates, because of extensive hydrogen bonding and π-π interactions between PDA and GO [7,33,34]. The AFM image for GDE microaggregates was shown (Figure 1d) which was similar to those of image presented by TEM (Figure 1c).

The blending of E and D was monitored using a UV spectrophotometer at about 480 nm (Figure 1e(A,B)). Most of the changes in absorption might were associated with the degree of polymerization of DA to dark red intermediates of PDA polymers [37]. The size of the DE complexes increased with the blending time of E and DA because polymerization of PDA continued. The blending time of E and DA was thus adjusted to 0.5~1 h to avoid the production of large aggregates of DE complexes (Figure 1a). The size of GDE microaggregates increased to 1.70 ± 0.01 μm (n = 3), which was approximately six times that of GO (~280 nm) (Figure 1c). Since the molecular weight of HRP is about ~44 kDa, which substantially exceeds that of DA, the PDA polymers might be located on the shell or outside layer of HRP (Figure 1a), protecting E from the harmful environment. To produce a GDES film, the GDE microaggregate suspension was homogeneously mixed with SF solution by strongly stirred, and then casted and dried. To produce a stable GDES film in water, the film was further treated in ethanol to induce β-sheets of SF (e.g., around 44% β-sheets induced after immersed at 60~85% of ethanol) [26] to avoid SF to be resolved in water.

### 3.2. ATR-FTIR Spectroscopic and SEM Analyses of GDES Films

**Figure 2:**
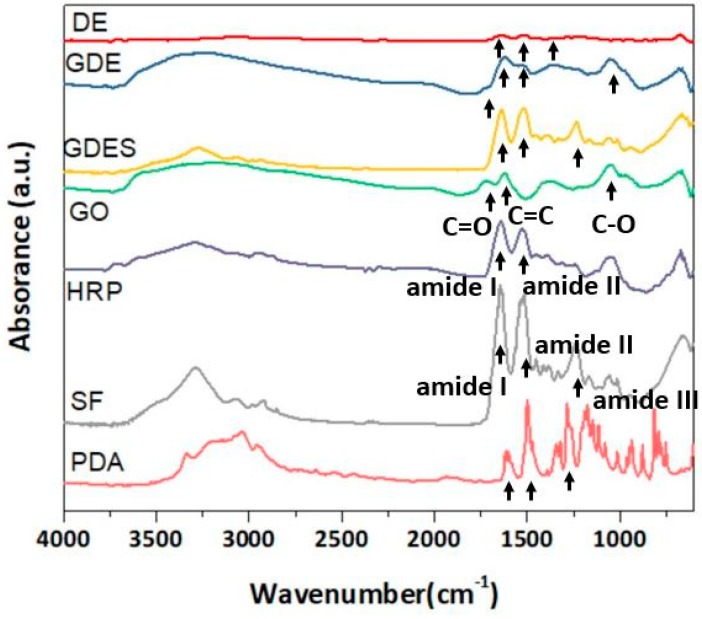
ATR-FTIR absorption spectra of varying composites of GDES films including DE, GDE, GDES, GO, HRP (E), PDA (D), and SF (S) were shown. The characteristics of the spectra of GDES films were mainly influenced by SF such as amide I, II and III since they contained large amounts of SF.

ATR-FTIR absorbed spectra were obtained to qualitatively examine the functional groups of the components in GDES films (Figure 2). The characteristic peaks of GO at 1723 cm^−1^, 1619 cm^−1^, and 1040 cm^−1^ were attributed to the stretching of the C=O, C=C, and C–O bonds of GO, respectively [38,39]. In the spectra of E (or HRP), the characteristic peaks were amide I, II (1526 cm^−1^) and III [40], which differed slightly from those associated with the amide bonds (of OR in) SF. In the spectra of PDA, the characteristic peaks at 1603 cm^−1^, 1499 cm^−1^, and 1283 cm^−1^ corresponded to the C=C stretching, and C=N stretching of the indole ring, and the C–O stretching of the primary amine, respectively [41]. The characteristic peaks of SF were at 1642 cm^−1^, 1515 cm^−1^, and 1229 cm^−1^, corresponding to amide I, amide II, and amide III, respectively (Figure 2) [26,42]. The characteristic peaks of the GDE microaggregates corresponded to amides I and II, and the C–O bond (~1040 cm^−1^), which were associated with E and G, respectively. Interestingly, the characteristic peak of GO at 1723 cm^−1^ was much lower in the spectra of DE and GDE owing to the partial reduction of GO to rGO [43,44]. The characteristic peaks of a GDES film corresponded to amides I, II and III, and the C–O bond, which were associated with SF, and G. respectively. Notably, most of characteristic peaks in SF were observed in the spectra of GDES films, possibly because the GDES films contained large amount of SF especially after ethanol treatment.

**Figure 3:**
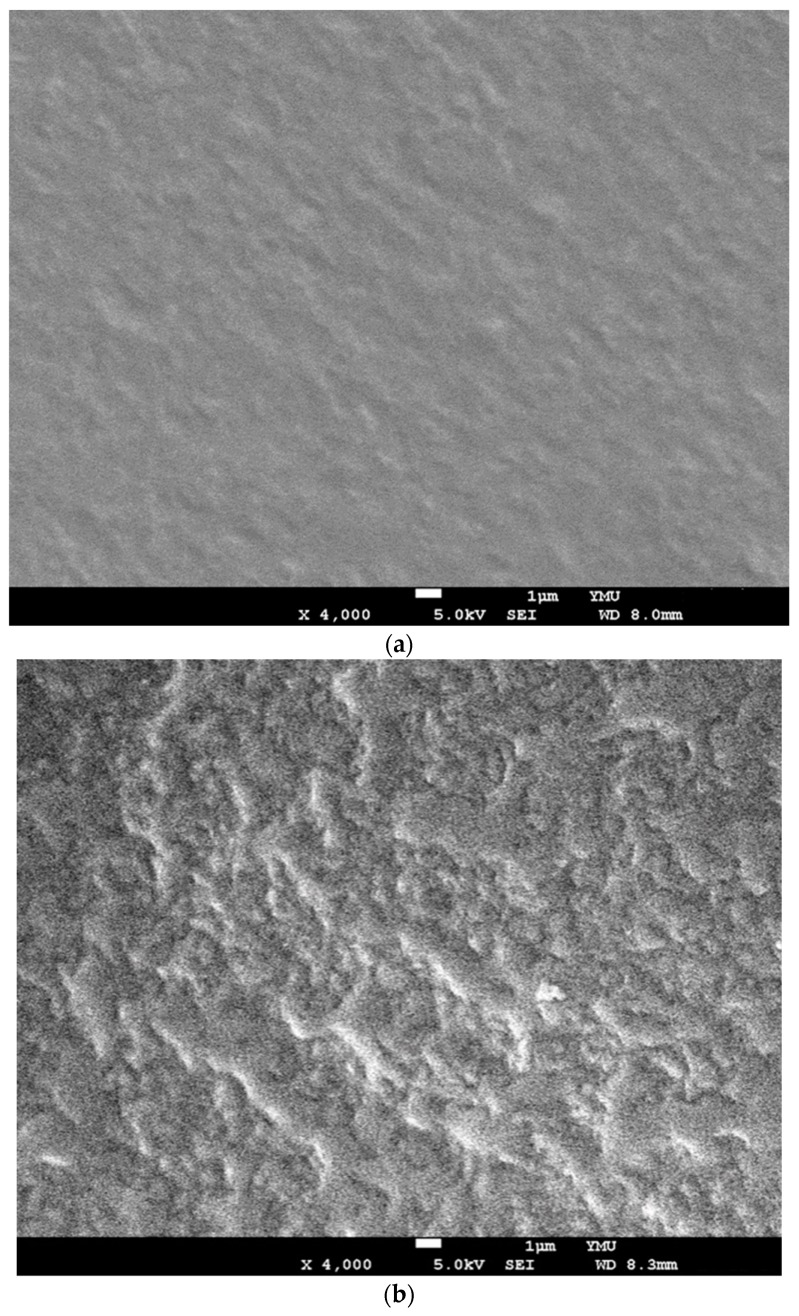

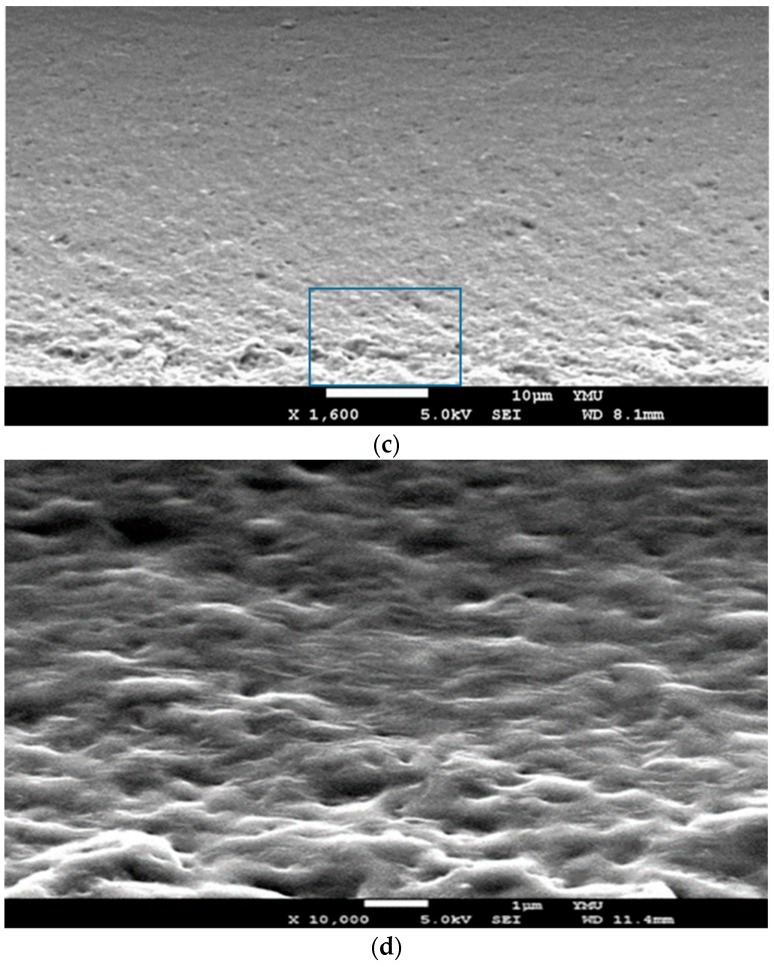
(**a**) SEM micrograph for the surfaces of GDES films (×4k), (**b**) SEM micrograph for the surfaces of GDES films after they scavenged H_2_O_2_ (×4k). (**c**) The SEM of cross-section of the GDES films after they were used to scavenge H_2_O_2_ are shown. (**d**) Shows high magnitude of micrograph at the bottom area of center region in Figure 3c.

SEM micrographs of the surfaces of GDES films before and after they were used to scavenge H_2_O_2_ are displayed (Figure 3a,b). The GDES film that had not scavenged H_2_O_2_ was relatively smooth (Figure 3a) while that of the film that had scavenged H_2_O_2_ was very rough and wrinkled (Figure 3b), possibly as a result of the formation many dityrosine bonds in S, induced by H_2_O_2_/HRP, which would have caused polymerization and the de-arrangement of polymers of S in the GDES film, producing the wrinkled surface (Figure 3b). However, the data to quantitate and prove many dityrosine bonds formations in the GDES films induced by H_2_O_2_/HRP was not able to be obtained in this study while the indirect evidence for those formations could be evaluated by the intensity of UV-excitable blue fluorescence of GDES films (Figure 3a) and reports elsewhere [20]. The SEM image of cross-section of the GDES films after they were used to scavenge H_2_O_2_ are shown (Figure 3c,d). Figure 3d showed high magnitude of micrograph at the bottom area of center region in Figure 3c. According to those Figure 3b,d, the morphologies of cross-section of GDES films after they scavenged H_2_O_2_ were rough and wrinkled which might be associated with the surfaces of the film (Figure 3b). Interestingly, using EDS to analyze the elements of surfaces (C, N and O) of the two aforementioned films (Figure 3a,b) revealed no difference in their elemental contents. For example, for GDES films that had not and had scavenged H_2_O_2_ the C, N, and O contents were 56.9 ± 0.2, 19.7 ± 0.6 and 23.5 ± 0.7% (n = 3); and 56.8 ± 2.4%, 20.3 ± 1.7 and 22.8 ± 0.7 (n = 3), respectively. Since EDS analysis for the image of film surfaces was semi-quantitative, the data for C, N, O analysis of the films for pre- and post- scavenged of H_2_O_2_ might qualitatively not be influenced.

### 3.3. H_2_O_2_-Scavenging by GDES Films

**Figure 4:**
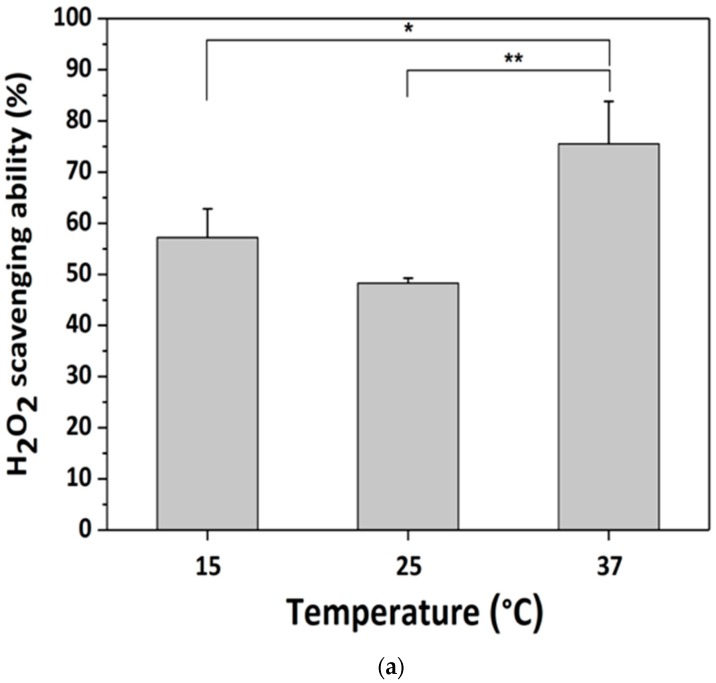

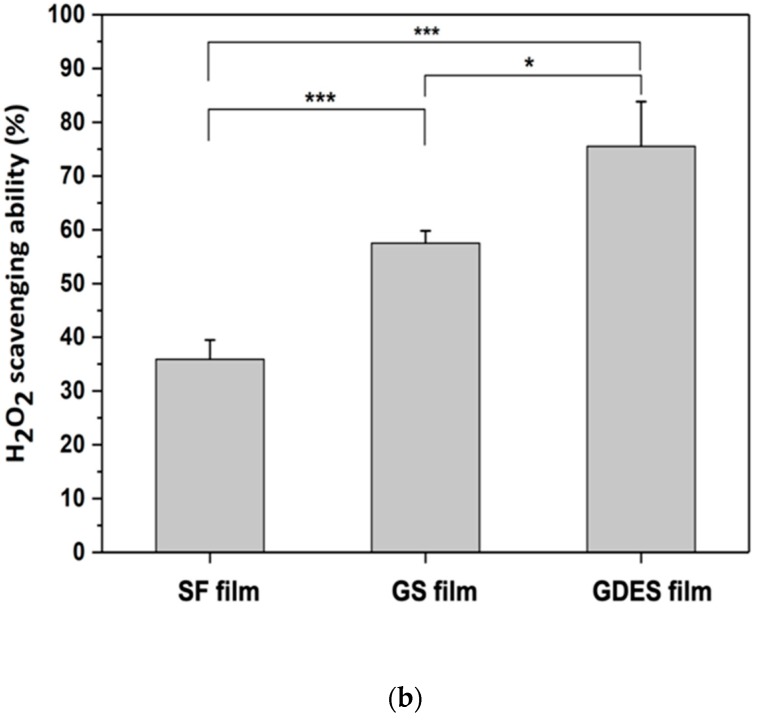
H_2_O_2_ scavenging ability tested by using 1, 10-phenanthroline agent to measure the residual H_2_O_2_ after immersed GDES films fully scavenged H_2_O_2_ in the solution. The 0% scavenging ability was defined as the measurement VIS values using the agent to examine the residual H _2_O_2_ without adding any film to scavenge H_2_O_2_, (**a**) H_2_O_2_ scavenging ability for GDES films at various temperatures, and (**b**) H_2_O_2_ scavenging ability for various films at 37 °C. The ability of GDES films was the highest compared with that for SF and GS films at 37 °C. (Note: * *p* < 0.05, ** *p* < 0.01, *** *p* < 0.001; Data are mean ± SD, n = 3).

Since the GDES films might be applied for sensing and scavenging H_2_O_2_ in outdoor at low temperature environments, the temperature might be one of the factors to affect the performance of the films. To quantify whether the scavenging abilities of GDES, GS and SF films were influenced by temperature, they were immersed in H_2_O_2_ solutions for 30 min at various temperatures. Scavenging ability was determined as the initial concentration of H_2_O_2_ in solution subtracted the residual H_2_O_2_ concentration in solution, as determined using 1,10-phenanthroline/FeCl_2_ method. Figure 4a presents the H_2_O_2_ scavenging ability of GDES films at various temperatures. It was highest, 73.5 ± 8.7% or 9.39 × 10^−2^ μmol H_2_O_2_ at 37 °C (n = 3) compared with the performance at low temperatures. The effect of temperature on H_2_O_2_ scavenging ability was consistent with reports that the activity of HRP is highest at approximately 35 °C [45,46]. Since the H_2_O_2_ scavenging ability of a GDES film was better in at 37 °C than that at low temperature, the photothermal properties of the film in order to raise working temperature to have a good sensing and scavenging ability were further investigated in the later section (Section 3.5). However, the factors which caused low H_2_O_2_ scavenging ability of the film at 25 °C needed to be further studied. Interestingly, SF films had an H_2_O_2_ scavenging ability of ~30% (Figure 4b), partly because they highly facilitated the self-decomposition of H_2_O_2_ into H_2_O and oxygen within 30 min because 1,10-phenanthroline/FeCl_2_ method needed to take around 30 min for determining the concentration of residual H_2_O_2_ in solution. Interestingly, the presence of SF in H_2_O_2_ solution increased the decomposition of H_2_O_2_ to H_2_O by approximately 12~30% although this increase depended on both the concentrations of SF (1 or 2%) and the mixing time (for example.10 min herein) of the SF and H_2_O_2_ solutions. The entrapment of H_2_O_2_ by the hydroxyl group, amine, and carboxyl group in SF may play a role in its scavenging (Figure 4b) [47]. However, the H_2_O_2_-scavenging ability of GS films exceeded that of SF (Figure 4b). Hence, whether GO plays a role in H_2_O_2_ scavenging in a GS film must be further investigated. Since the H_2_O_2_ scavenging ability of PDA was reported [19], the greater H_2_O_2_-scavenging ability of the GDES film might arise from the synergistic effects of scavenging H_2_O_2_ by its reduction to H_2_O by HRP and the radical character of the catechol/quinone structure in the PDA structure. Figure 1b displays the assumed mechanisms of H_2_O_2_ scavenging by GDES film. H_2_O_2_ molecules in solution are assumed to diffuse through void spaces among SF polymers to the inner components of the GDES film, where they are reduced by E to H_2_O. The amount of H_2_O_2_ that is reduced by a GDES film can be used to evaluate the ability of the film to scavenge H_2_O_2_ (Figure 1b).

### 3.4. Use of UV-Excitable Blue Fluorescence of GDES Films to Probe H_2_O_2_

**Figure 5:**
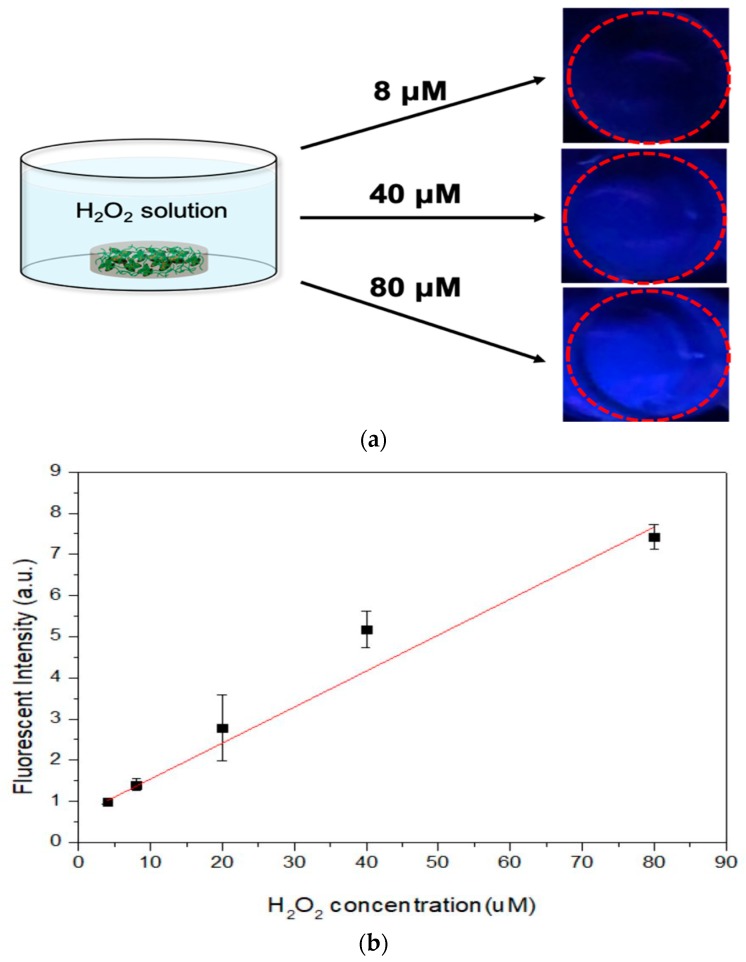
(**a**) Images of increasing blue fluorescent intensities of supernatants. Images of the fluorescence (marked in red circles) increased with increasing H_2_O_2_ concentrations (e.g., 8, 40 and 80 μM) in solutions, (**b**) The normalized blue fluorescent intensities after GDES films fully scavenged H_2_O_2_ were linearly increased with increasing H_2_O_2_ concentration from the range of 4.0 to 80 μM (Data are mean ± SD, n = 3; R^2^ = 0.984).

The reduction reactions of H_2_O_2_ in aqueous solution by E in a GDES film may trigger simultaneous oxidation reactions of tyrosine in S in the film to produce tyrosyl radicals, ultimately forming dityrosine bonds in S, which emit blue fluorescence under UV irradiation (Supplementary Figure S1 and Figure 5a), such that the GDES film can serve as a naked eye-probe of H_2_O_2_ [23]. The absorbance of blue fluorescence (at 425 nm) verse the reaction times would reach stable at 30 min till the time of the end observation (e.g., 60 min) and the values at 30 min were chosen in this study (Figure S1). The linearity between the intensity of the blue fluorescence of the dityrosine bonds of SF that was leak from GDES films and the initial concentration of H_2_O_2_ in aqueous solution was determined (Figure 5b, n = 3). The normalized intensity of blue fluorescence was linearly correlated with the initial concentration of H_2_O_2_ in aqueous solution from 4.0 to 80 μM (R^2^ = 0.984%, Figure 5b, n = 3) with a detection limit of 4 μM although the deviations of data at H_2_O_2_ concentration of 40 μM were needed to further be investigated. H_2_O_2_ concentration. In aqueous solution has frequently been determined from the fluorescence intensity or absorbance using fluorescent dyes or chromogenic reagents, such as Amplex^®^ red [14] and 3,5,3′,5′-tetramethylbenzidine (TMB) [15]. However, neither dye is biocompatible, so each may have cytotoxic effects at the site of application such as a wound, limiting its biomedical application in situ. Also, none of those chromogenic reagents can locally scavenge sufficient H_2_O_2_. Although blue fluorescence of dityrosine bonds of SF, induced by UV, wasbeen reported [23], this investigation is the first to determine the H_2_O_2_-scavenging ability of GDES films, and quantify the linearity between the intensity of their blue fluorescence and the H_2_O_2_ concentration in aqueous solution. Although similar UV-excitable fluorescence system using SF, HRP and H_2_O_2_ in liquid state was reported by the author’s group [48], there were two major differences between two studies; 1. The enzyme, HRP, was immobilized in PDA and coated by SF to produce a film in this study while all reactants in the early system were reacted in a liquid state, and 2. the concentrations of H_2_O_2_ herein was in uM level while the early one was mM, revealing there were 10^3^ differences [49]. The detection range of H_2_O_2_ concentrations herein was suitable for detecting harmful concentrations in vivo (10^0^~10^2^ μM) [1,49,50]. However, the mechanisms of complex electron transfers among HRP, PDA, and SF in a GDES film that are involved in scavenging and probing H_2_O_2_ in aqueous solution were not investigated herein. These mechanisms will be investigated in the near future. However, without the presence of HRP in the film, for instance, S and GS films, it is hardly to stimulate the rates of the reduction reactions of H_2_O_2_ to H_2_O which might result in producing small amounts of dityrosine bonds formations in those films. There is hardly or not able to detect the intensity of UV-excitable blue fluorescence for the S and GS films.

### 3.5. Photothermal Responses of GDES Films

**Figure 6:**
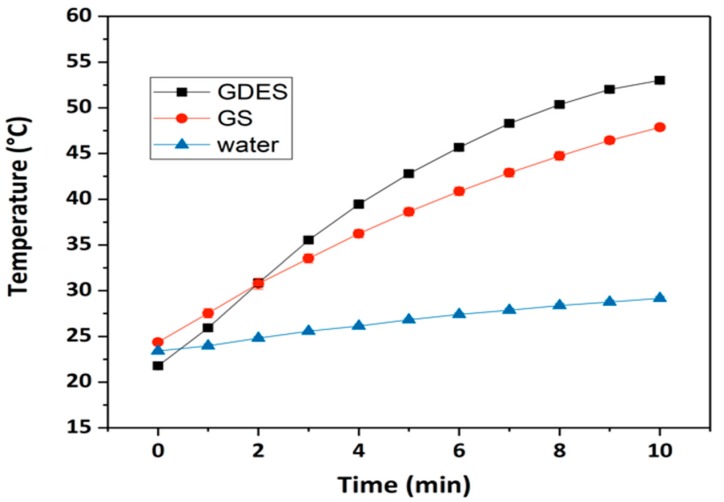
Photothermal responses of GDES or GS films were carried out by heating the films in water by 2W and at 808 nm NIR for 10 min. The temperature of aqueous increased shortly for GDES or GS films. The temperature of water is about the same for 10 min heating. (Data are mean ± SD, n = 3).

Since the GDES films might be applied for scavenging and sensing H_2_O_2_ in outdoor at low temperature environments, raising the working temperature might affect the performance of the films. The photothermal property of GO is widely documented [8]. Figure 6 plots the photothermal responses of GDES films during 10 min of NIR irradiation. The temperature of the GDES-containing solution increased from 22 °C to 52 °C whereas that of the GS-containing solution increased from 24 °C to 48 °C. The temperatures of the solutions that contained GDES films and GS films significantly exceed that of the solution without those films under irradiation by NIR, revealing that the GO in the system exhibited a favorable photothermal response whereas the PDA in the GDES films made a minor contribution to it. These findings probably follow from the low PDA content in the GDES films because the proportions of GO and PDA in the GDES films were about 1.6 wt% and 0.6 wt%, respectively. Notably, the photothermal response of a GDES film can be used to heat samples in situ under NIR irradiation, enabling the working temperature to be optimally adjusted for the enzyme, E, in the film to enhance scavenge locally H_2_O_2_. Therefore, the property of scavenging H_2_O_2_ locally of the films could be carried out in outdoor at low temperature. The sensing local H_2_O_2_ by the films might be improved at the working temperature near 37 °C because the activity of HRP is highest at approximately 35 °C [47,48].

### 3.6. In Vitro Biocompatibility of GDES Films

**Figure 7:**
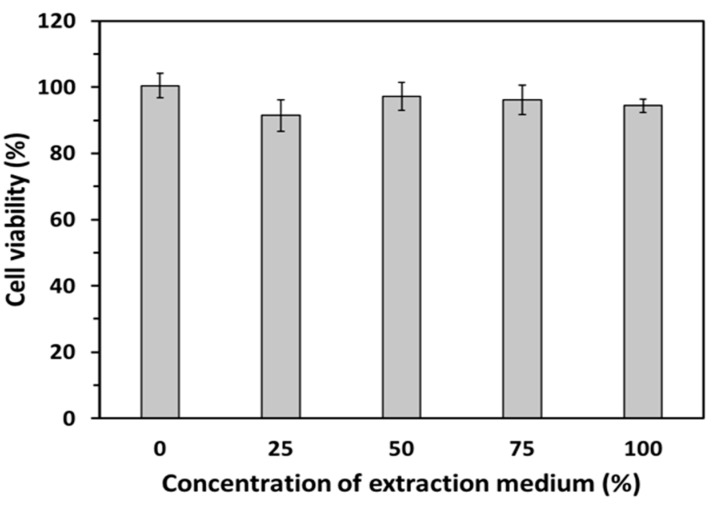
Cell viability of L929 fibroblasts were performed to examine the biocompatibility of GDES films which were incubated with various percentages (e.g., 25~100%) of extract medium from GDES films/incubated medium according to the ISO-10993-5. Accordingly, the GDES films were biocompatible. (Data are mean ± SD, n = 3).

The viabilities of L929 fibroblasts that were incubated with various extraction solutions which were taken from cultural mediums in which GDES films had been immersed for 24 h were determined and shown in Figure 7 (n = 3). The viabilities of L929 fibroblasts that were incubated in the group of 100% extraction solution remained around 90%, which was slightly lower than that of the other groups. According to ISO 10993-5, the GDES films were therefore non-toxic biomaterials and suitable for use in in vitro and further in vivo studies. Since these films mainly comprise SF and PDA, their biocompatibility might be reasonable [19,26]. Although GO was reported to be a cytotoxic material because it has negative surface charges with the possible production of reactive oxygen species (ROS) on its surface [51,52], its surface might have been fully covered by DE complexes and S in this study. Therefore, the cytotoxic factors on the GO surface might have been suppressed by other components of the GDES film, and the cytotoxicity of GO and the film might have been negligible (Figure 7).

## 4. Conclusions

In this work, photothermally responsive GDES films that can scavenge and probe H_2_O_2_ by UV-excitable blue fluorescence was developed. The TEM micrographs and AFM topographies for GDE aggregates (about 2 μM) were shown. In addition, the rough and wriggle surfaces of SEM micrographs for the GDES films after they scavenged H_2_O_2_ were shown (Figure 3b). The H_2_O_2_-scavenging abilities of the GDES film were influenced by temperature with the highest value of 9.39 × 10^−2^ μmol H_2_O_2_ per film at 37 °C (Figure 4a). The normalized blue fluorescent intensities after GDES films scavenged H_2_O_2_ were linearly increased with increasing H_2_O_2_ concentration from the range of 4.0 to 80 μM (Figure 5b). In addition, the GDES film exhibited a photothermal response and good biocompatibility (Figure 7). Since GDES films could be used to heat samples in situ under NIR irradiation, and enabled raising the working temperature thereof to 37 °C for enhancing scavenging H_2_O_2_ locally. Therefore, the property of scavenging H_2_O_2_ locally of the films could be carried out in outdoor at low temperature. In addition, GDES films may have the potential for locally scavenging associated with probing H_2_O_2_ under various pathological conditions, such as those that pertain to un-healing wound management.

## Figures and Tables

**Table 1:** Abbreviation of nomenclature.

Abbreviation	
G	GO, Graphene oxide
D	PDA, Polydopamine
E	HRP, Horseradish peroxidase
S	SF, Silk fibroin
DE	PDA-HRP complex
GS	GO covered by SF
GDE	GO-PDA/HRP microaggregates
GDES	GO-PDA/HRP microaggregates covered by SF

## Supplementary Materials

The following are available online at https://www.mdpi.com/1424-8220/20/2/366/s1.
