# Supplementary material for: Developing a Silk Fibroin Composite Film to Scavenge and Probe H2O2 Associated with UV-Excitable Blue Fluorescence
Source: Sensors (Basel). 2020 Jan 8;20(2):366. doi: 10.3390/s20020366 (PMC7014260; doi:10.3390/s20020366)
Supplement: Supplementary file 1 [file sensors-20-00366-s001.pdf]

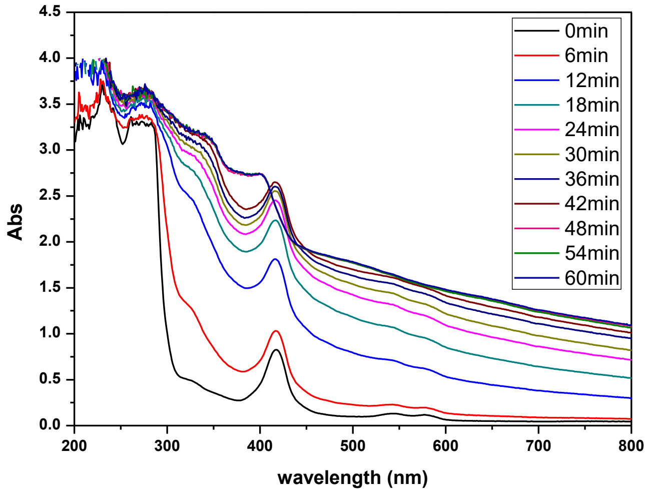

Figure S1. The absorbance of blue fluorescence (at 425 nm) verse the times of SF/HRP/H_2O_2 reaction. The absorbance of blue fluorescence would reach stable from 30 min till the time of the end observation (e.g., 60 min.). and the values at 30 min were chosen in this study.
